# Activity of Various Cathepsin Proteases and Enrichment of Klotho Protein in the Urine and Urinary Extracellular Vesicles After SARS-CoV-2 Infection

**DOI:** 10.3390/v17010025

**Published:** 2024-12-28

**Authors:** Niharika Bala, Ramish H. Rafay, Sarah C. Glover, Abdel A. Alli

**Affiliations:** 1 Department of Medicine, Division of Nephrology, Hypertension, and Renal Transplantation, University of Florida College of Medicine, Gainesville, FL 32608, USA; niharikabala@ufl.edu (N.B.); rafay.r@ufl.edu (R.H.R.); 2 Department of Physiology and Aging, University of Florida College of Medicine, Gainesville, FL 32608, USA; 3 Department of Medicine, Tulane University School of Medicine, New Orleans, LA 70112, USA; sglover3@tulane.edu

**Keywords:** cathepsins, alpha-klotho, COVID-19, SARS-CoV-2

## Abstract

**Background:** The severe acute respiratory syndrome coronavirus-2 (SARS-CoV-2) is responsible for causing the Coronavirus disease 2019 (COVID-19) outbreak. While mutations cause the emergence of new variants, the ancestral SARS-CoV-2 strain is unique among other strains. **Methods:** Various clinical parameters, the activity of cathepsin proteases, and the concentration of various proteins were measured in urine samples from COVID-19-negative participants and COVID-19-positive participants. Urinary extracellular vesicles (uEVs) were isolated from urine samples from the two groups and used for proteomic analysis and subsequent pathway analyses. **Results:** Activity levels of cathepsin S and L were greater in the urine of COVID-19-positive participants. The concentration of C-reactive protein, transmembrane serine protease 2, and klotho protein were significantly greater in the urine of COVID-19-positive participants. There was a greater amount of uEVs in the COVID-19 group and klotho protein was found to be enriched in uEVs from the COVID-19 group. Pathway analyses of the proteomics data showed most of the identified proteins were involved in signal transduction, stress response, protein metabolism, and transport. The identified proteins were predominantly associated with cellular membranes and with function of the cytoskeleton, enzyme regulation, and signal transduction. **Conclusions:** Taken together, our data identify novel urinary biomarkers that could be used to further investigate the long-term effects of SARS-CoV-2 infection.

## 1. Introduction

The severe acute respiratory syndrome coronavirus type 2 (SARS-CoV-2) that causes coronavirus disease 2019 (COVID-19) relies on the activity of host proteases for virus entry. SARS-CoV-2 is subject to pre-activation by furin-mediated cleavage of the spike (S) protein [1]. In addition, trypsin-like proteases have been shown to activate the S protein of SARS-CoV-2. Yamazaki et al. used a pseudotyped VSV bearing the S protein of SARS-CoV-2 to demonstrate that trypsin increases SARS-CoV2 infectivity [2].

Several studies have shown that other types of proteases are involved in SARS-CoV-2 entry into host cells [1,3,4]. Studies have shown that the virus enters host cells in a pH- and receptor-mediated endocytosis-dependent mechanism that involves the cleavage and activation of the spike protein by lysosomal cathepsin proteases. A study by Nie et al. showed that the expression of several different cathepsins including cathepsin B, D, E, H, K, L, S, and Z are upregulated in the lungs of COVID-19 patients [5]. The metalloproteases ADAM10 and ADAM17 have been shown to cleave the SARS-CoV-2 spike protein, contribute to priming of the S protein, and promote cell entry of the virus [6].

The S protein of SARS-CoV-2 was reported to reduce the availability of cytoprotective proteins including Klotho protein [7]. Alpha-Klotho is expressed in various tissues including the kidney. Soluble alpha-Klotho is a product of proteolytic cleavage of the transmembrane alpha-Klotho protein [8]. The two cleavage sites for ADAM10 and ADAM17 that have been identified include one side close to the transmembrane region and another between the KL1 and KL2 domains [9]. The cleavage of Klotho allows for the release of the endocrine product into biological fluids including the blood, cerebrospinal fluid, and urine. Alpha-Klotho in the serum has been considered as a severity marker for critically ill COVID-19 patients and found to show moderate diagnostic potential [10]. Data from a study by Alem et al. suggest that exogenous administration of Klotho protein offers survival benefits in a mouse model of COVID-19 [11]. Another study by Xu et al. showed the therapeutic potential of a Klotho-derived peptide 1 in mitigating SARS-CoV-2-associated acute kidney injury [12].

To test our hypothesis that specific cathepsins and extracellular vesicle (EV) enriched proteins can serve as urinary biomarkers after COVID-19 infection, we used urine samples from COVID-19-negative participants and COVID-19-positive participants to measure the activity of various cathepsin proteases and isolate urinary EVs (uEVs) for downstream proteomics analysis. In addition, we provide a discussion of the significance of these findings.

## 2. Materials and Methods

### 2.1. COVID-19-Negative Participants and COVID-19-Positive Participants

A total of 37 urine samples from COVID-19-positive participants of the ancestral variant and a total of 25 urine samples from COVID-19-negative participants collected before 2019 were used in this study. Age and gender of the COVID-19-negative and COVID-19-positive participants are listed in Supplementary Table S1. All samples were collected from consented participants under an approved IRB (#2020-0065).

### 2.2. Osmolality

An Osmometer Model 3320 was used to determine the osmolality of human urine samples isolated from COVID-19-negative participants and COVID-19-positive participants. The urine samples were centrifuged, and supernatant was collected. The probe was cleaned, and the sample tip was loaded. A total of 30 µL of sample was loaded onto the probe and inserted into the machine.

### 2.3. Nitric Oxide Assay

All the reagents of the kit were brought to room temperature and prepared as per the manufacturer’s instructions. The urine samples were centrifuged to collect the supernatant, and the assay was performed according to the manufacturer’s instructions (Abcam 212517, Waltham, MA, USA).

### 2.4. C Reactive Protein

Urinary C reactive protein concentrations were determined in both COVID-19-negative participants and COVID-19-positive participants following the manufacturer’s instructions provided with the C reactive protein ELISA kit (Millipore Sigma, RAB0096, Burlington, MA, USA).

### 2.5. TMPSSR2 Assay

A single step ELISA was performed following the manufacturer’s instructions (Abcam, ab283552, Waltham, MA, USA) to determine the levels of TMPSSR2 in the urine samples from both groups. Urine samples were centrifuged, and the supernatant was used for the assay.

### 2.6. Creatinine Assay

Centrifuged urinary samples were diluted at 1:20 in ultrapure 1× PBS (filtered using a 0.2 µm Nalgene filter (Thermo Fisher Scientific, Waltham, MA, USA) and were then used to determine the creatinine levels following the manufacturer’s instructions (Abcam, ab204537).

### 2.7. Albumin Assay

All the urine samples were subjected to centrifugation and the supernatant was collected. This supernatant was used to determine the urinary albumin concentration while following the manufacturer’s instructions (Proteintech, KE00076, Rosemont, IL, USA).

### 2.8. EV Isolation

Urine samples totaling 10 mL from each human subject were, first, centrifuged at 1000× *g* for 15 min at 4 °C. Next, a 0.22 µm rapid-flow Nalgene filter (Thermo Fisher Scientific, Waltham, MA, USA) was used to filter the supernatant. The filtrate underwent ultracentrifugation at 52,000 RPM for 90 min at 4 °C using a fixed-angle Ti-70 rotor (Beckman Coulter, Inc., Brea, CA, USA). The final extracellular vesicle pellet was resuspended in 250 μL of ultrapure 1× PBS (Thermo Fisher Scientific, Waltham, MA, USA) and then stored at −20 °C.

### 2.9. Nanoparticle Tracking Analysis

The urinary extracellular vesicles (uEVs) isolated by the ultracentrifugation method were analyzed for size and concentration using an NS300 device, connected to NTA 3.4 Build 3.4.4 Software (Malvern, Malvern, UK). A 1:1000 dilution of the uEVs in filtered 1× PBS was injected into the system, with an automatic infusion pump delivering the samples at a flow rate of 40 and at room temperature.

### 2.10. Western Blotting and Densitometric Analysis

Isolated uEVs from COVID-19-negative participants and COVID-19-positive participants were lysed prior to Western blotting as previously described by our group [13] with the following modifications. The lysed EVs were loaded onto 4–20% Tris-Glycine gels (Thermo Fisher Scientific, Waltham, MA, USA) and separated at 200 volts for 1 h using a BioRad Criterion electrophoresis system (Hercules, CA, USA) before being transferred onto nitrocellulose membranes (Thermo Fisher Scientific, Waltham, MA, USA) using a BioRad Criterion transfer system, blocked, and incubated with primary antibodies (Table 1), followed by incubation with secondary antibody and then ECL reagent. ImageJ software [14] was used to quantify the band intensity.

### 2.11. Transmission Electron Microscopy

Urinary EVs isolated from COVID-19-negative participants and COVID-19-positive participants were resuspended in ultrapure 1× PBS solution before 25 µL of this suspension was added to 25 µL of 4% paraformaldehyde solution. For each uEV sample, a formvar carbon grid was carefully floated on top of a 10 µL drop of uEV sample placed on a sheet of parafilm and left to absorb for 20 min at room temperature. Each grid was then transferred to a 50 µL drop of filtered 1× PBS, and then transferred to a 50 µL drop of 1% glutaraldehyde 1× PBS solution before being incubated for 5 min at room temperature. Afterwards, each grid underwent a series of 8 washes in 50 µL distilled water for 2 min intervals at room temperature. Each grid was then floated on top of a 50 µL drop of uranyle-oxalate mixture (1:1 of 0.15 M oxalic acid and 4% aqueous uranyl acetate, pH 7) while being covered and incubated for 5 min at room temperature. Each grid was then incubated with 50 µL methyl cellulose-uranyle acetate solution for 10 min in the dark before the solution was blotted on Whatman filter paper number 1 and the grids were left to air dry for 10 min. A Hitachi H7600 transmission electron microscope (Hitachi Technologies, Clarksburg, MD, USA) was used to image the grids.

### 2.12. Cathepsin Protease Assays

Urine samples were subjected to centrifugation at 1000× *g* for 10 min and the supernatant was used for the activity assays. Cathepsin B, S, L, and D activity assays were performed using urine samples of the COVID-19-negative participants and COVID-19-positive participants following the manufacture’s protocol (abcam, ab65300, ab65307; ab65306; ab65302, Waltham, MA, USA).

### 2.13. Protein Digestion and Nano-LC/MS/MS Based Proteomics

Proteins were harvested in ice cold EasyPep™ MS buffer (Thermo Fisher Scientific, Waltham, MA, USA). After qubit analysis, equal amounts of protein (6 µg total) were used per sample, and volumes were adjusted to 100 µL with the EasyPep lysis buffer. Next, 50 µL of reduction and alkylation solution were added to each sample and incubated at 95 °C for 10 min. After cooling, 50 µL of the reconstituted enzyme was added and samples were digested at 37 °C for 1.5 h. Afterwards, 50 µL digestion stop solutionwas added, and the samples were run through peptide clean-up columns to remove detergents and salts. The sample eluent was dried via speedvac and reconstituted in 50 µL 0.1% formic acid in water for analysis.

Nano-liquid chromatography tandem mass spectrometry (Nano-LC/MS/MS) was performed using a Q Exactive HF Orbitrap mass spectrometer (Thermo Scientific, Waltham, MA, USA) equipped with an EASY Spray nanospray source (Thermo Scientific Waltham, MA, USA) and operated in positive ion mode. The liquid chromatography was carried out on a UltiMate™ 3000 RSLCnano system (Thermo Fisher, Waltham, MA, USA). The mobile phase A consisted of water containing 0.1% formic acid and the mobile phase B was made of acetonitrile with 0.1% formic acid. The mobile phase A for the loading pump consisted of water and 0.1% trifluoracetic acid. Five mL of each sample was injected into a PharmaFluidics mPACä C18 trapping column (C18, 5 mm pillar diameter, 10 mm length, 2.5 mm inter-pillar distance) at a 10 mL/mL flow rate. This was held for 3 min and washed with 1%B to desalt and concentrate the peptides. Afterwards, the peptides were eluted off the trap onto the column. Chromatographic separations were performed using C18 columns of 5 mm pillar diameter, 50 cm length, 2.5 mm inter-pillar distance. The column temperature was maintained at 40 °C and a flow rate of 750 nL/min was maintained for the initial 15 min, after which it was decreased to 300 nL/min. Initially, a gradient of 1%B to 20%B over 100 min followed by 45%B over 20 min (for a total run time of 150 min) was used to elute peptides directly off the column into the Q Exactive system.

All MS/MS spectra were analyzed using the Chimerys node (Thermo Fisher Scientific, Waltham, MA, USA; Proteome Discoverer 3.0.1.27). Chimerys was set up to search Homo sapiens (NcbiAV TaxID = 9606) (v2023-01-24) and Universal Protein Contaminants fasta [15] assuming the digestion enzyme trypsin. Chimerys was searched with a fragment ion mass tolerance of 0.020 Da and a precursor ion tolerance of 10.0 ppm. Carbamidomethyl of cysteine was specified in Chimerys as a fixed modification.

Precursor ion intensity label-free quantitation was performed using Proteome Discoverer (Thermo Fisher Scientific vs 3.01.1.27). The two groups (your groups) were compared using a “non-nested” study factor. Normalization was derived by using all peptides. Protein abundances were calculated by summed abundances, meaning the protein abundances are calculated by summing sample abundances of the connected peptide groups. Fisher’s exact test (pairwise ratio-based) was used to calculate *p*-values with low intensity resampling value imputation included. Adjusted *p*-values were calculated using Benjamini–Hochberg.

### 2.14. Statistical Analysis

For analysis of the proteomics dataset, a Fisher’s exact test (pairwise ratio-based) was used to calculate *p*-values with low intensity resampling value imputation. Adjusted *p*-values were calculated using Benjamini–Hochberg. Sigmaplot 15.0 software was used for analysis and data plotting. A student *t*-test was used to determine the statistical significance between the two groups. A one-way ANOVA was performed to make comparisons between multiple groups. A *p*-value less than 0.05 was considered significant.

## 3. Results

### 3.1. ACR and Urinary Osmolality, CRP, and NO Levels in COVID-19-Negative Participants and COVID-19-Positive Participants

Common urinary parameters were measured in urine samples collected from COVID-19-negative participants and COVID-19-positive participants. An albumin to creatinine (ACR) ratio was comparable between the COVID-19-positive participants and the COVID-19-negative participants (Table 2). Urinary nitric oxide levels were significantly greater in the COVID-19-negative participant group while urinary C-reactive protein (CRP) levels were significantly greater in the COVID-19-positive participant group (Table 2). Urinary osmolality was comparable between the two groups (Table 2).

### 3.2. Activity of Cathepsin Proteases in the Urine of COVID-19-Negative Participants and COVID-19-Positive Participants

**Figure 1:**
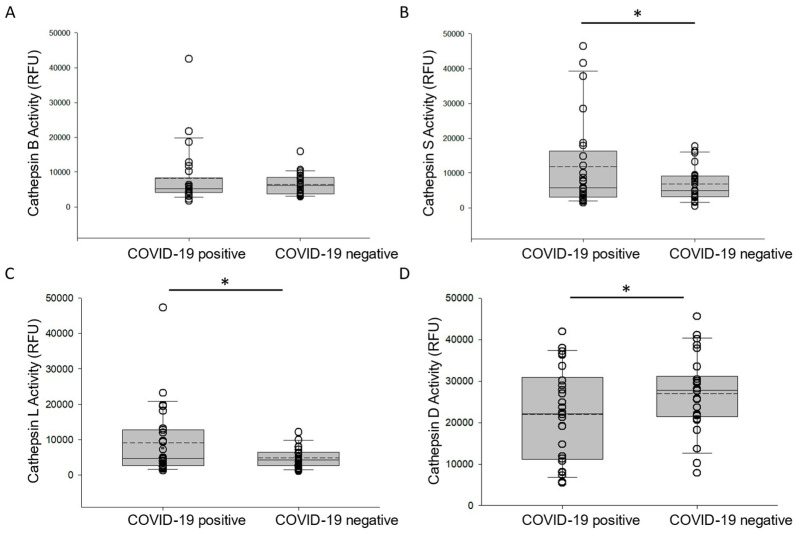
Activity of various cathepsins in urine samples from COVID-19-negative participants and COVID-19-positive participants. (**A**) cathepsin B activity, (**B**) cathepsin S activity, (**C**) cathepsin L activity, and (**D**) cathepsin D activity in urine samples from COVID-19-negative participants and COVID-19-positive participants. Box and whisker plots show the mean (dotted line) and median (solid line) for the data points. * represents a *p*-value < 0.05. n = 25 samples per group. RFU refers to relative fluorescence units.

Multiple studies have shown that lysosomal proteases, including various members of the cathepsin family, are involved in the mechanism of SARS-CoV-2 entry into host cells. We investigated whether there are significant differences in the activity of various cathepsin proteases in urine samples from COVID-19-negative participants and COVID-19-positive participants. As shown in Figure 1B,C, the activity of cathepsin S and cathepsin L were greater in the urine of the COVID-19-positive participant group compared to the COVID-19-negative participant group. Conversely, the activity of cathepsin D was lower in the urine of the COVID-19-positive participant group (Figure 1D) and the activity of cathepsin B was comparable between the two groups (Figure 1A).

### 3.3. Increased TMPSSR2 in the Urine of COVID-19-Positive Participantsp

**Figure 2:**
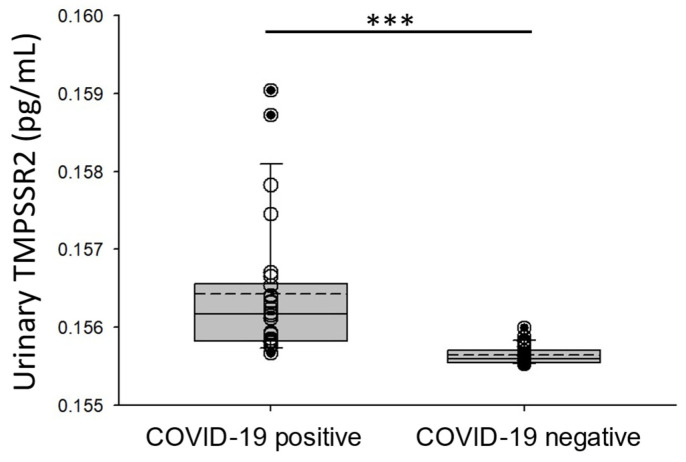
ELISA of TMPSSR2 concentration in urine samples from COVID-19-negative participants and COVID-19-positive participants. Box and whisker plot show the mean (dotted line) and median (solid line) for the data points. *** represents a *p*-value < 0.001. n = 25 samples per group.

Since the transmembrane serine protease 2 (TMPRSS2) and angiotensin-converting enzyme 2 (ACE2) proteins are involved in SARS-CoV-2 viral entry [16], we investigated whether TMPRSS2 was elevated in the urine of COVID-19-positive participants compared to the urine of COVID-19-negative participants. As shown in Figure 2, there was a greater concentration of TMPRSS2 in the urine of the COVID-19-positive participants compared to the control group.

### 3.4. Increased Klotho Concentration in the Urine of COVID-19-Negative Participants and COVID-19-Positive Participants

**Figure 3:**
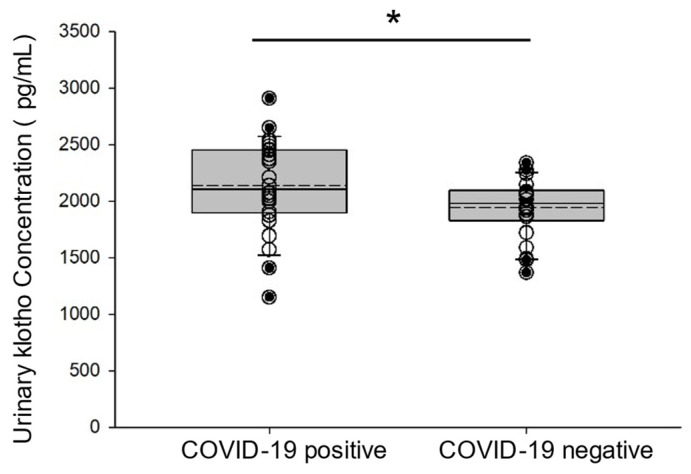
ELISA of Klotho concentration in urine samples from COVID-19-negative participants and COVID-19-positive participants. Box and whisker plot show the mean (dotted line) and median (solid line) for the data points. * represents a *p*-value < 0.05. n = 25 samples per group.

Urinary Klotho has been reported to serve as a possible early biomarker of acute kidney injury [17]. Klotho is cleaved by the same metalloproteases that cleave and prime the SARS-CoV-2 spike protein for cellular entry. Therefore, we measured the concentration of Klotho in the urine of COVID-19-negative participants and COVID-19-positive participants. Klotho concentration was found to be significantly elevated in urine samples from the COVID-19-positive participant group compared to the control group (Figure 3).

### 3.5. Concentration of EVs in the Urine of COVID-19-Negative Participants and COVID-19-Positive Participants

**Figure 4:**
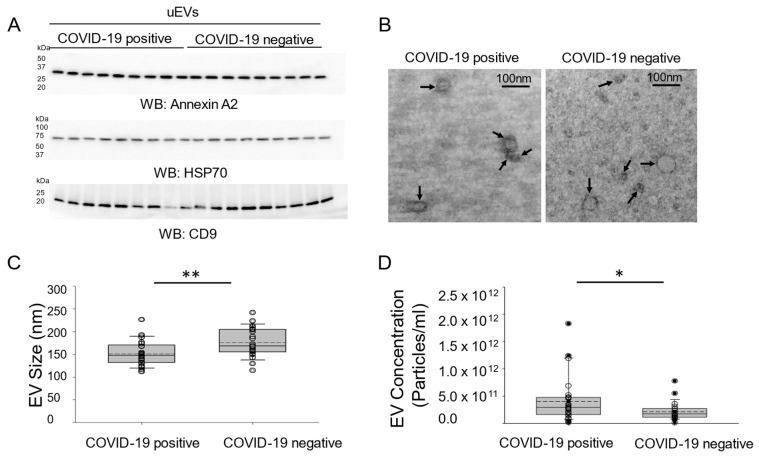
Characterization of uEVs isolated from COVID-19-negative participants and COVID-19-positive participants. (**A**) Western blot analysis of the uEV markers annexin A2, HSP70, and CD9; n = 9 per group; (**B**) representative transmission electron microscopy micrographs of uEV preparations from the COVID-19-negative and COVID-19-positive participant groups; (**C**) nanoparticle tracking analysis showing a difference in size of the uEV preps from the two groups; and (**D**) nanoparticle tracking analysis showing a difference in concentration of the uEV preps from the two groups. Box and whisker plots show the mean (dotted line) and median (solid line) for the data points. * represents a *p*-value < 0.05. ** represents a *p*-value < 0.01; n = 25 samples per group.

Based on previous studies by our group [13], and additional studies (Supplementary Figure S1), uEVs not subject to multiple freeze–thaws maintain their integrity. uEVs are considered a useful biomarker for kidney injury and other kidney-associated diseases. COVID-19-positive participants frequently develop new kidney disease or worsening of kidney disease, which may result in an increase in EVs being filtered and excreted in the urine. Here, we characterized uEVs isolated from urine samples of COVID-19-negative participants and COVID-19-positive participants by Western blotting for different uEV markers, transmission electron microscopy, and nanoparticle tracking analysis. Common uEV biomarkers including annexin A2, HSP70, and CD9 were all present in each uEV preparation from the COVID-19-negative and COVID-19-positive participant groups, and the levels of each biomarker were generally comparable between the two groups (Figure 4A). Transmission electron microscopy analysis (Figure 4B) and nanoparticle tracking analysis (Figure 4C) of uEVs showed that there is a mixed population of EVs ranging in sizes between the two groups. Additionally, nanoparticle tracking analysis showed that there was a greater amount of uEVs in the COVID-19-positive participant group compared to the COVID-19-negative participant group (Figure 4D).

### 3.6. Increased Concentration of Klotho in uEVs from COVID-19-Negative Participants and COVID-19-Positive Participants

**Figure 5:**
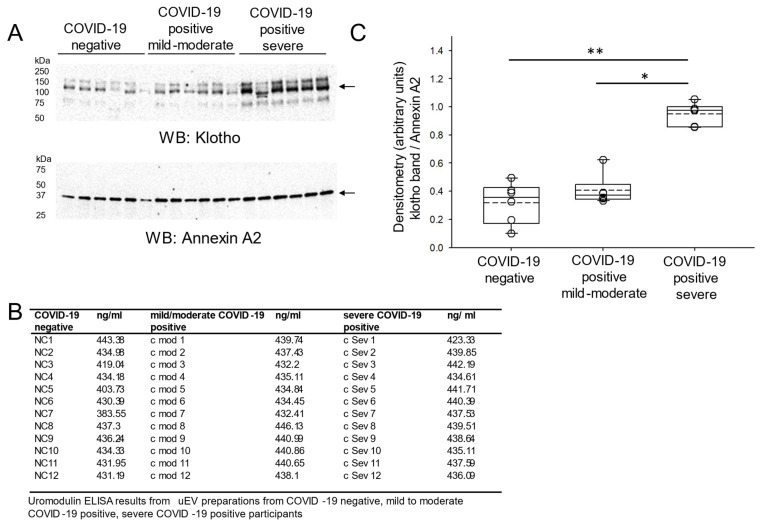
Western blot and densitometric analysis of klotho protein in uEVs from COVID-19-negative participants and COVID-19-positive participants. (**A**) Representative Western blot showing Klotho protein enrichment (top blot) in uEVs from COVID-19-negative participants, mild-to-moderate COVID-19-positive participants and severe COVID-19-positive participants. Representative Western blot of Annexin A2 protein enrichment (bottom blot) in these samples from the same blot was used to assess lane loading. (**B**) Table of uromodulin concentration in uEV preparations from COVID-19-negative participants, mild-to-moderate COVID-19-positive participants, and severe COVID-19-positive participants. (**C**) Densitometry analysis of Klotho protein enriched in uEVs normalized to annexin A2 protein expression. Box and whisker plots show the mean (dotted line) and median (solid line) for the data points. * represents a *p*-value < 0.05, ** represents a *p*-value < 0.01. n = 12 samples per group.

A recent study reported a direct relationship between the hypoxia-inducible protein 1 (HYOU1, ORP150) and clinical characteristics of COVID-19 severity [18]. We performed an ELISA for ORP150 using urine samples from the COVID-19-negative participants and COVID-19-positive participants and grouped the COVID-19-positive participant samples into mild-to-moderate-severity and severe disease groups based on the ORP150 levels. Since we identified increased Klotho protein in the urine of COVID-19-positive participants compared to COVID-19-negative participants, we next investigated whether Klotho protein is enriched in uEVs from the COVID-19 group. Compared to uEVs isolated from the COVID-19-negative group and the COVID-19-positive mild-to-moderate severity group, Klotho protein enrichment was greater in the COVID-19-positive severe group where densitometric analysis was performed and Annexin A2 was used to assess lane loading (Figure 5).

### 3.7. Proteomic Analysis of uEVs from COVID-19-Negative Participants and COVID-19-Positive Participants

**Figure 6:**
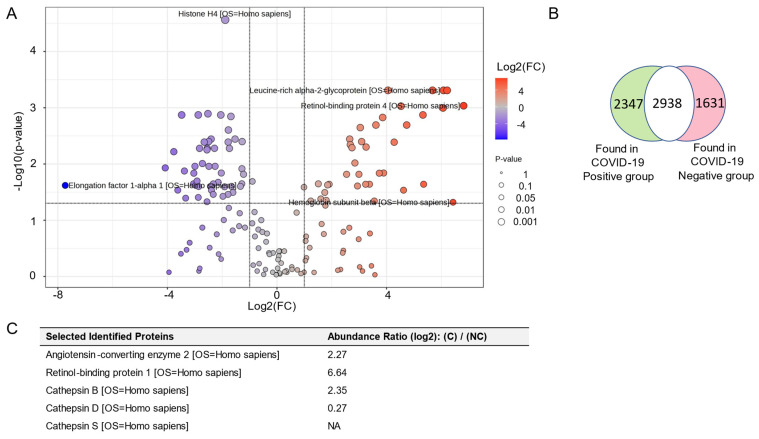
Proteomic analysis of uEVs isolated from COVID-19 and non-COVID-19 patients. (**A**) Volcano plot of differentially expressed peptides/proteins in the COVID-19-negative participant and COVID-19-positive participant groups; the intensity bar on the right represents the degree of fold change of COVID-19-positive/COVID-19-negative and the size of the circles represents the significance of the fold change. MetaboAnalyst 6.0 software [19] was used to create the Volcano plot. (**B**) Venn diagram showing the number of peptides/proteins exclusively enriched in each group or common between the two groups. (**C**) Table showing a list of selected identified proteins enriched in uEVs from COVID-19-positive participants compared to uEVs from COVID-19-negative participants. The fold change threshold was set to 2.0 and the *p*-value threshold was set to 0.05.

A proteomic analysis showed peptides/proteins that were differentially enriched in the uEVS from the COVID-19-positive and COVID-19-negative groups (Figure 6A). A total of 2347 peptides/proteins were exclusively enriched in the uEVs from the COVID-19-positive participant group and 1631 peptides/proteins were enriched in the COVID-19-negative group (Figure 6B). Among the peptides/proteins enriched in the COVID-19-positive group were multiple members of the cathepsin family including cathepsin B, cathepsin D, and cathepsin S (Figure 6C). The proteomic dataset DOI link: 10.6084/m9.figshare.26838763 (https://figshare.com/s/5aabde80811c56734275, accessed on 26 August 2024) identifies proteins enriched in uEVs from each of the two groups.

### 3.8. Pathway Analysis of the uEV Isolated from COVID-19-Negative Participants and COVID-19-Positive Participants

**Figure 7:**
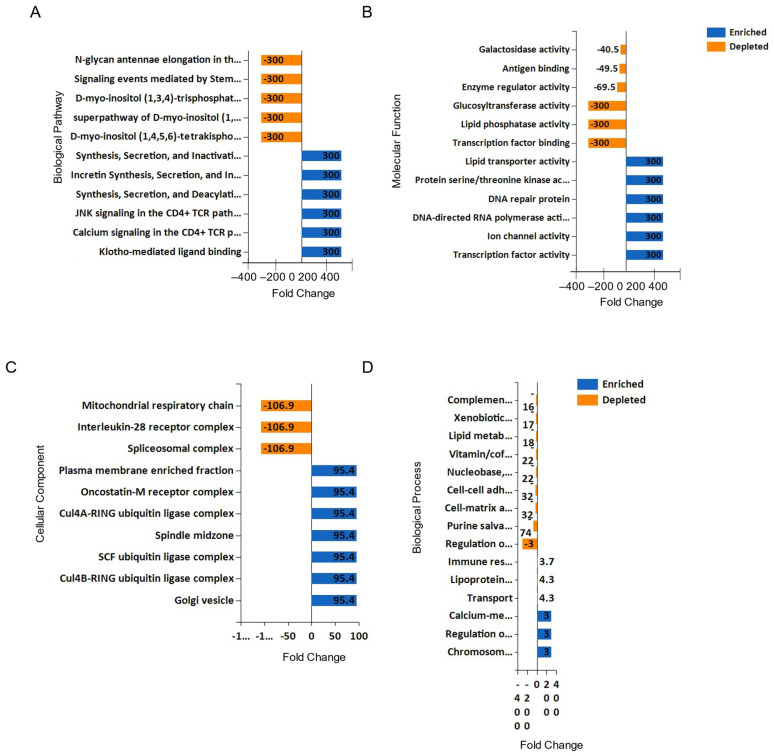
Bioinformatic analysis of the proteomic dataset from uEVs of COVID-19-negative participants and COVID-19-positive participants. (**A**) Biological pathway, (**B**) molecular function, (**C**) cellular component, and (**D**) biological process. n = 9 per group.

A majority of the identified proteins exclusive to either of the two groups are involved in signal transduction, stress response, protein metabolism, transport, and cell organization (Figure 7). In addition, these proteins are mainly associated with the plasma membrane (Figure 7). The molecular functions of these identified proteins include signal transduction, enzyme regulation, nucleic acid binding, and cytoskeleton dynamics (Figure 7).

## 4. Discussion

To our knowledge, this is the first proteomic study that compares the proteins enriched in uEV between COVID-19-negative participants and COVID-19-positive participants. Numerous studies have investigated proteins enriched in serum EV between COVID-19-negative participants and COVID-19-positive participants in different contexts. For example, studies have compared EV proteins in plasma EVs between recovered COVID-19 patients and healthy controls [20] and the plasma EV proteome between mild and severe COVID-19 patients [21], and characterized the landscape of circulating EV proteomics and phosphoproteomics for hospitalized patients with COVID-19 and type 2 diabetes mellitus [22]. Since no study has investigated differences in uEVs isolated from COVID-19-positive participants compared to uEVs isolated from COVID-19-negative participants in correlation with the enrichment of cathepsin proteases, we performed a proteomic analysis of differentially enriched protein/peptides in uEVs from the two groups. In our proteomic dataset, we saw an increased abundance of cathepsin proteases B, D, F, and Z in the urinary EVs of COVID-19-positive participants. The largest difference was seen with cathepsins Z and B with abundance ratios on Log2 scale of 4.1 and 2.35, respectively. Cathepsins D and E represent the two aspartic cathepsins and cathepsins A and G represent the two serine cathepsins that are expressed in humans [23]. Cysteine cathepsins including cathepsins B, C, F, H, K, L, V, O, S, W, and X represent the largest family of cathepsins that are expressed in humans [23]. Specific cathepsin proteases play an important role in priming cellular entry of the SARS-CoV-2.

Alpha-Klotho is a pleiotropic protein that is mainly expressed as a transmembrane protein and a soluble form in renal epithelial cells of the kidney tubule. The soluble form of alpha-Klotho results from the cleavage of the membrane-bound form [8,24]. Alpha-Klotho is found in biological fluids including serum and urine. Hu et al. showed that the kidney clears alpha-Klotho from the blood and allows its excretion into the urine [25]. Klotho was reported to regulate transporters and ion channels via autocrine- and paracrine-dependent mechanisms. Multiple groups showed that Klotho upregulates the epithelial calcium channel TRPV5 [26,27,28]. In another study, Cha et al. showed that Klotho increases the cell-surface expression of ROMK [29]. Another study showed that Klotho downregulates the renal sodium-dependent phosphate cotransporter (NaPi-IIa) [30]. Multiple studies have investigated alpha-Klotho as a biomarker in cardiovascular disease [31,32] and renal disease [33,34]. Hu et al. reported that alpha-Klotho is unstable in human urine and suggested that optimal measurements of alpha-Klotho should be performed using fresh catheter urine [25].

In chronic kidney disease, urinary Klotho levels seem to be inversely related to urinary klotho excretion and have also been demonstrated to be involved in sodium homeostasis. An increase in urinary Klotho excretion can result in a decline in serum Klotho levels and an increase in sodium wasting [35]. Declining levels of Klotho are associated with the vascular calcifications seen in chronic kidney disease [36]. This effect is attenuated with Klotho exposure [37]. In the context of COVID-19, Klotho has been shown to have a protective effect on the kidneys of mice infected with SARS-COV2. Mice deficient in Klotho had a higher likelihood of developing COVID-19-induced acute kidney injury and Klotho-derived peptide 1 attenuated the viral-induced tubular injury [12].

While a study by Nie et al. explored the organ-specific proteome in deceased COVID-19-positive participants, and identified dysregulated proteins and related pathways, our study centers around measuring specific cathepsin and TMPRSS2 protease activities in urine and Klotho protein in uEVs as a novel biomarker for assessing COVID-19 severity.

The results of our study are consistent with those of other studies. For example, Medeiros et al. showed that there is a greater concentration of EVs in the urine of hospitalized adult COVID-19 patients compared to healthy volunteers [38]. Our proteomic data analyzing the packaged cargo of uEVs are consistent with that of other published studies. For example, a study by Vergara et al. reported that urinary angiotensin-converting enzyme 2 (ACE2) is increased in COVID-19 patients [39]. We show that uEVs from COVID-19-positive participants are enriched in ACE2 compared to COVID-19-negative participants (DOI link: 10.6084/m9.figshare.26838763 (https://figshare.com/s/5aabde80811c56734275, accessed on 26 August 2024). A study by Karras et al. reported elevated levels of urinary retinol binding protein in COVID-19 patients [40]. Our proteomic dataset also identified an increase in retinol binding protein in uEVs from COVID-19-positive participants (DOI link: 10.6084/m9.figshare.26838763 (https://figshare.com/s/5aabde80811c56734275, accessed on 26 August 2024).

Some limitations of our study include us not sorting the uEVs isolated from the cohort of COVID-19-negative participants and COVID-19-positive participants to determine the relative amount of uEVs from the systemic circulation that were filtered, the EVs that were secreted, or the EVs that were released from renal tubular cells. A second limitation is that we did not measure whether the activity level of endogenous protease inhibitors is altered in urine samples and in uEVs from COVID-19-negative participants and COVID-19-positive participants. A third limitation was that there could be potential batch effects due to it not being feasible to collect urine samples from each patient in the different groups at the same time and perform each of the various assays for every patient sample separately at the time of urine collection.

Future studies may investigate whether these cathepsins play a role in either the dysregulation or activation of the NLRP3 inflammasome in different organs systems after COVID-19 infection. Studies have shown that SARS-CoV-2 causes NLRP3 inflammasome activation in microglia [41], endothelial cells [42], and neutrophils [43].

## Figures and Tables

**Table 1:** Antibodies and assays used in this study.

Antibody/Assay	Company	Catalogue Number	Application
Annexin A2	Cell Signaling Tech	8235	Western Blotting
HSP70-HRP	BioLegend	648005	Western Blotting
CD9	Abcam	ab223052	Western Blotting
Klotho	Santa Cruz	sc-515939	Western Blotting
Albumin Elisa	Proteintech	KE00076	Urinary Assay
Creatinine Assay	Abcam	ab204537	Urinary Assay
Human Klotho Duo Set	R & D Systems	DY5335-05	Urinary Assay
C-Reactive Protein ELISA	Millipore Sigma	RAB0096	Urinary Assay
Cathepsin B	Abcam	ab65300	Urinary Assay
Cathepsin S	Abcam	ab65307	Urinary Assay
Cathepsin D	Abcam	ab65302	Urinary Assay
Cathepsin L	Abcam	ab65306	Urinary Assay
Nitric oxide assay	Abcam	ab212517	Urinary Assay
TMPSSR2 ELISA	Abcam	ab283552	Urinary Assay

**Table 2:** Urinary parameters from COVID-19-negative participants and COVID-19-positive participants. N = 25 for each group.

Assay	MEAN ± SEM		*p*-Value (*t*-Test)
	COVID-19 Positive	COVID-19 Negative	
Albumin–Creatinine Ratio	3.393 ± 0.184	3.045 ± 0.196	0.0764
Nitric oxide (µM)	0.0186 ± 0.00189	0.0253 ± 0.000550	0.00212
C-Reactive Protein (pg/mL)	4.208 ± 0.56	2.491 ± 0.08	0.0054
Osmolality (mosm/kg)	514.308 ± 32.799	480.846 ± 36.433	0.4979

## Data Availability

The datasets will be made available upon reasonable request by contacting the corresponding author.

## Supplementary Materials

The following supporting information can be downloaded at: https://www.mdpi.com/article/10.3390/v17010025/s1, Figure S1: (A) Nanoparticle tracking analysis showing a representative uEV profile of COVID-19-negative participants and COVID-19-positive participant samples without multiple freeze–thaws. (B) Nanoparticle tracking analysis showing a representative uEV profile of a COVID-19-negative participant and COVID-19-positive participant samples with multiple freeze–thaws. Table S1: COVID-19-negative and -positive participant age and gender.
