# Supplementary material for: Activity of Various Cathepsin Proteases and Enrichment of Klotho Protein in the Urine and Urinary Extracellular Vesicles After SARS-CoV-2 Infection
Source: Viruses. 2024 Dec 28;17(1):25. doi: 10.3390/v17010025 (PMC11768607; doi:10.3390/v17010025)
Supplement: Supplementary file 1 [file viruses-17-00025-s001.zip › viruses-3210027-supplementary.pdf]

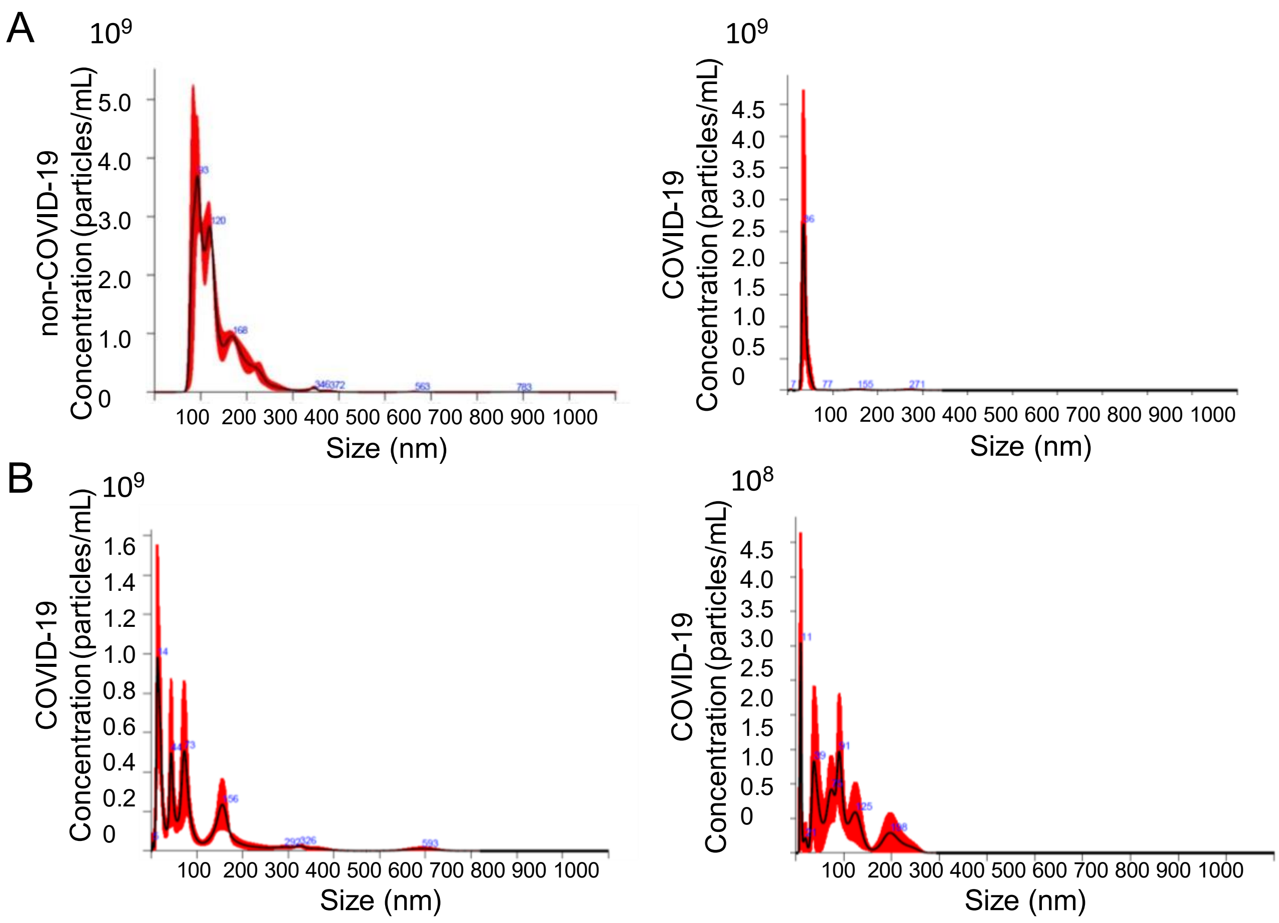

Supplementary Figure S1. (A) Nanoparticle tracking analysis showing a representative uEV profile of COVID-19 negative participants and COVID-19 positive participant samples without multiple freeze thaws. (B) Nanoparticle tracking analysis showing a representative uEV profile of a COVID-19 negative participant and COVID-19 positive participant samples with multiple freeze thaws.

	Mean Age	Variant	Gender
COVID-19 negative	53	NA	16 females, 9 males
COVID-19 positive mild/ moderate	53	Ancestral	17 females, 6 males
COVID 19 positive severe	59	Ancestral	7 females, 8 males

Supplementary Table S1. COVID-19 negative and positive participant age and gender.
